# Identification of MicroRNAs and Their Targets Associated with Fruit-Bagging and Subsequent Sunlight Re-exposure in the “Granny Smith” Apple Exocarp Using High-Throughput Sequencing

**DOI:** 10.3389/fpls.2016.00027

**Published:** 2016-02-01

**Authors:** Dong Qu, Fei Yan, Rui Meng, Xiaobing Jiang, Huijuan Yang, Ziyi Gao, Yonghui Dong, Yazhou Yang, Zhengyang Zhao

**Affiliations:** ^1^College of Horticulture, Northwest A&F UniversityYangling, China; ^2^Shaanxi Province Key Laboratory of Bio-Resources, Shaanxi University of TechnologyHanzhong, China; ^3^Department of Plant and Environmental Sciences, Weizmann Institute of ScienceRehovot, Israel; ^4^Apple E&T Research Centre of Shaanxi ProvinceYangling, China

**Keywords:** apple, bagging treatment, anthocyanin, high-throughput sequencing, microRNA, target gene expression

## Abstract

Bagged fruits of green apple cultivar “Granny Smith” have been found to turn cardinal red after debagging during fruit-ripening in the Loess Plateau region of China. To understand this phenomenon at post-transcriptional level, we have investigated the roles of microRNAs (miRNAs) in response to debagging. Three small RNA libraries were primarily constructed from peels of “Granny Smith” apples subjected to bagging followed by sunlight re-exposure treatments (0, 6 h, 1 day) (debagging), and from peels of apples without any bagging treatments (0, 6 h, 1 day). 201 known miRNAs belonging to 43 miRNA families and 220 novel miRNAs were identified via high-throughput sequencing. Some miRNAs were found to be differentially expressed after debagging, which indicated that miRNAs affected anthocyanin accumulation through their target genes in mature apple. To further explore the effect of debagging on miRNAs regulating the expression of anthocyanin regulatory genes, four miRNAs and their target genes regulating anthocyanin accumulation, miR156, miR828, miR858, and miR5072, were compared between green cultivar “Granny Smith” and red cultivar “Starkrimson.” Results showed that mdm-miR828 and mdm-miR858 regulated anthocyanin contents in both apple cultivars, while mdm-miR156 only affected anthocyanin accumulation in “Granny Smith,” and miR5072 affected anthocyanin accumulation in “Starkrimson.” Additional analysis of gene ontology for the differentially expressed miRNAs after debagging treatments and their predicted target genes showed that they were involved in photo-protective response after debagging from 0 h to 1 day; they might play important roles in fruit development and adaptation to high light stress.

## Introduction

Apple skin coloration depends on the concentration and distribution of anthocyanins, chlorophyll and carotenoids (Lancaster, 1992). The red color of apple skin is attributed to the presence of anthocyanins and flavonols. Usually abundant anthocyanins, in apple skin, provide essential cultivar differentiation for consumers and are implicated in the health attributes of apple fruits (Lancaster, 1992; Ma et al., 2014; Vimolmangkang et al., 2014). In addition, anthocyanins also play vital roles in plant-environment interactions, such as protection against drought, UV radiation, high light stress and disease (Van den Ende and El-Esawe, 2014).

Previous studies show that fruit-bagging can enhance accumulation of anthocyanins, thereby improving the appearance of apple fruit (Wang et al., 2013; Meng et al., 2015). Moreover, anthocyanin and flavonols content can be further increased in the bagged apples after subsequent debagging treatment due to increased light sensitivity of the apple fruits (Arakawa, 1988; Li and Cheng, 2008a; Chen et al., 2012). After debagging, the expression levels of structural genes encoding the enzymes involved in the anthocyanin biosynthesis pathway are enhanced by UV-B treatments (Ubi et al., 2006; Wang et al., 2013). Moreover, the expression level of *MdMYB1* transcript factors, regulator genes involved in anthocyanin biosynthesis pathway, have been also reported to be strongly light-inducible (Liu et al., 2013; Zhang et al., 2013).

**Figure 1:**
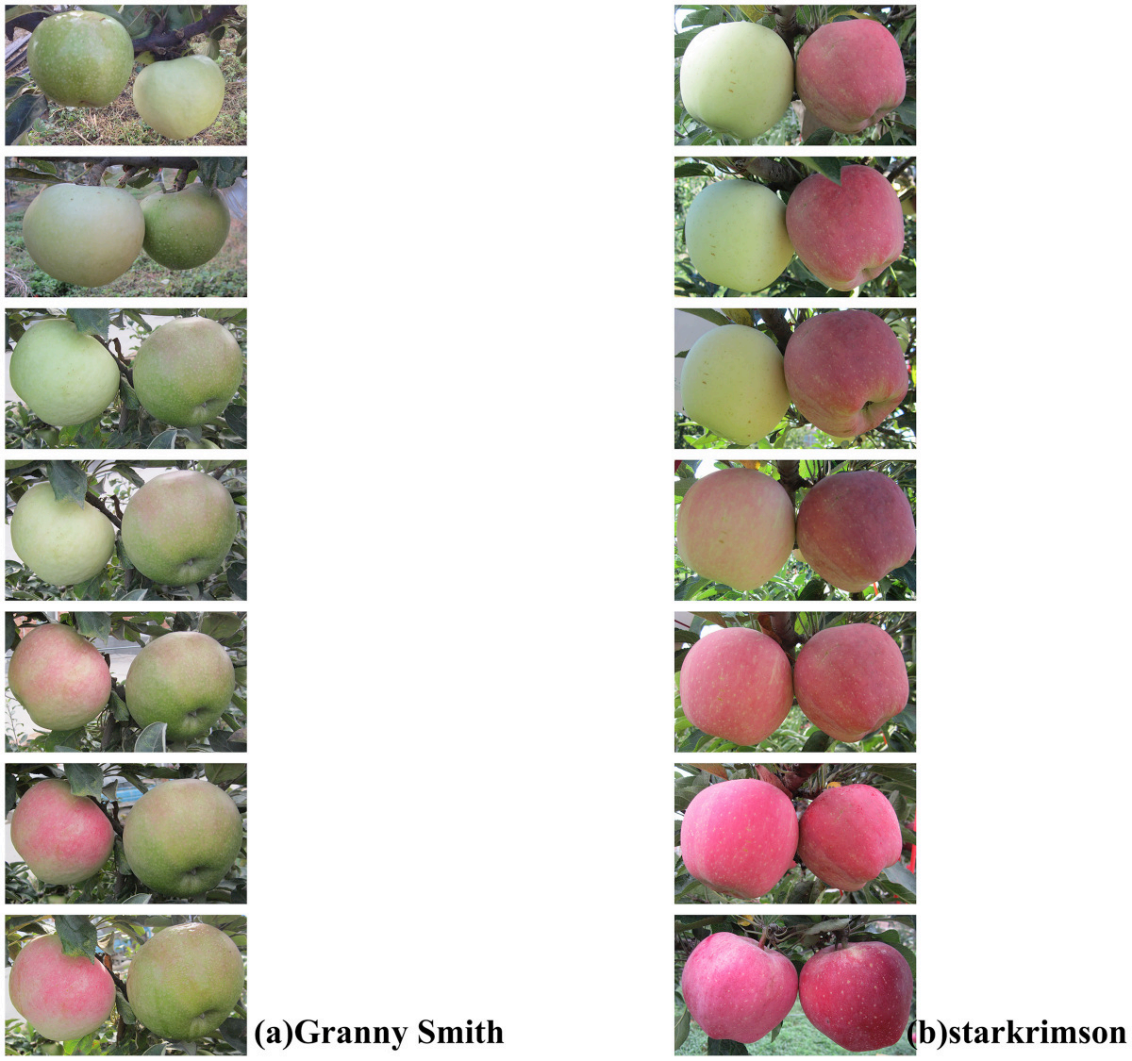
**Effect of debagging on fruit coloration of “Granny Smith” and “Starkrimson.” (A)** Non-bagged (left) and debagged “Granny Smith” (right) at 0 h, 1, 2, 4, 6, 8 days; **(B)**: Non-bagged (left) and debagged “Starkrimson” (right) at 0 h, 1, 2, 4, 6, 8 days. Note: In **(A)**, the fruits in the left and right side in the first picture are debagged and non-bagged “Granny Smith,” respectively.

An interesting phenomenon has been observed in the Loess Plateau region of China wherein bagged green cultivar “Granny Smith” can turn cardinal red after debagging during fruit ripening (Figure 1A; Liu et al., 2013; Wang et al., 2013). To date, several physiological and molecular mechanisms have been proposed to explain this coloration phenomenon. Studies show that cyanidin 3-galactoside is the most abundant anthocyanin in “Granny Smith” fruits, and it is significantly accumulated within 7 days after debagging (Liu et al., 2013; Wang et al., 2013). Expression of the structural genes involved in anthocyanin biosynthesis, *MdF3H, MdDFR, MdANS*, and *MdUFGT*, are significantly increased in “Granny Smith” apple skin after debagging (Wang et al., 2013; Zhang et al., 2013). Expression analysis of the *MdUFGT* gene family shows that *MdUFGT2* is only up-regulated in cultivar “Granny Simith” (non-red), while in contrast *MdUFGT4* is only up-regulated in red-skinned cultivar “Starkrimson” (Meng et al., 2015). Beyond structural genes, transcription factors involved in anthocyanin biosynthesis have also been investigated. *WD40, bHLH* (basic helix-loop-helix) and *MYB* are three families of transcriptional regulator genes involved in apple anthocyanin regulation. Transcription levels of *MdMYB10, MdMYB1*, and *MdBHLH33* are substantially up-regulated in “Granny Smith” after debagging (Wang et al., 2013; Zhang et al., 2013); the analysis of *MdMYB* regulatory gene families reveals that in comparison to *MdMYB1-1, MdMYB1-2* plays a crucial role in anthocyanin accumulation after debagging (Meng et al., 2015).

Mature microRNAs (miRNAs) are a class of small non-coding single-strand RNA molecules with 21–23 nt length. MicroRNAs are vital in post-transcriptional regulation, plant growth and development, signal transduction and stress responses (Sunkar et al., 2007; Axtell and Bowman, 2008). Some miRNAs can be induced by different stress environments such as UV-B radiation, cold and phosphate deficiency. For example, the expression levels of miRNAs are up-regulated when *Arabidopsis* plants are subjected to UV-B stress(Zhou et al., 2007). A miRNA-regulatory network (13 up-regulated miRNAs and 11 down-regulated miRNAs) under UV-B stress is identified in *Populus tremula* (Jia et al., 2009).

In addition, studies show that miRNAs can regulate coloration and anthocyanin accumulation through their target genes, while only a few miRNAs responsible for anthocyanin biosynthesis have been reported. In *Arabidopsis* stems, the increase of miR156 activity represses the functions of its target gene, *SPL* (squamosa promoter binding protein-like compounds), and promotes accumulation of anthocyanin, whereas the reduction of miR156 activity leads to the accumulation of flavonols (Gou et al., 2011). In addition, miRNA828 can regulate anthocyanin biosynthesis through its target gene *AtTAS4-siR81*(-). *AtTAS4-siR81*(-) gene can positively regulate anthocyanin accumulation through its target gene *MYB*s (Rajagopalan et al., 2006; Hsieh et al., 2009; Luo et al., 2012). Previous study shows that *MdTAS4-siR81*(-) has target genes similar to *AtTAS4-siR81*(-), and it targets a bHLH transcription factor, which interacts with *MdMYB10* to regulate anthocyanin biosynthesis in apple (Xia et al., 2012).

When bagged fruits are re-exposed to sunlight (debagging) the biosynthesis and accumulation of anthocyanin can be promoted in apple peel during fruit maturation. However, the effects of fruit-bagging and debagging on coloration of “Granny Smith” apple peel at post-transcriptional regulation level remain unknown. How the color formation of bagged “Granny Smith” after debagging is associated with miRNAs regulation and how miRNAs and target genes regulate anthocyanin biosynthesis are both still poorly understood. In this study, we have analyzed the changes of miRNAs expression levels in “Granny Smith” apple skin and predicted the target genes of candidate miRNAs after debagging treatment. In order to further identify potential miRNAs responsible for coloration of “Granny Smith” after debagging; we analyzed the expression levels of four candidate miRNAs and their target genes, which genes involved in regulating anthocyanin biosynthesis in “Granny Smith.” Finally, the expression levels of these candidate miRNAs and their target genes in green cultivar “Granny Smith” and in red cultivar “Starkrimson” were compared in order to confirm the regulatory roles of miRNAs in “Granny Smith.”

## Materials and methods

### Plant materials and treatments

Two apple cultivars (*Malus* × *domestica* Borkh.), green cultivar “Granny Smith” (6-year-old, grafted onto M26 rootstock) and red cultivar “Starkrimson” (6-year-old, grafted onto M26 rootstock), were planted in 4 × 2 m space at Baishui apple experimental station of Northwest A&F University (N35°21′and E109°55′, the Loess Plateau region of China), Shaanxi, China. Ten trees were used for each cultivar in our experiment; half of the fruits were used as treatment group and the other half as control group. In the treatment groups, “Starkrimson” and “Granny Smith” fruitlets were bagged with light-impermeable double-layer paper bags (Hongtai, Shaanxi, China) at 10 DAFB (days after full blown) and 40 DAFB, respectively. Unbagged apple fruits were used as control groups and they grew under natural sunlight condition. Before harvesting, “Starkrimson” and “Granny Smith” bagged fruits were debagged at 120 DAFB and 160 DAFB (15 days prior to harvest), respectively. The bagged fruits were harvested at 0, 6 h, 1, 2, 4, 6, and 8 days after debagging and each point-in-time sample consisted of 10 fruits (two fruits per tree). The sampling time of 0 h, 1, 2, 4, 6, and 8 days was at 8 a.m. for each day. Ten fruits from each control group at each corresponding time point were meanwhile collected. All samples were immediately stored in ice boxes after collection. Fruit peel (~1 mm in thickness) was collected with a peeler and samples from the same group were pooled. All samples were immediately frozen in liquid nitrogen and stored at −80°C for subsequent analysis.

### Anthocyanin analysis

Anthocyanin concentrations in “Granny Smith” and “Starkrimson” fruit peel were measured using the method described by Drumm-Herrel and Mohr (1982). Samples (0.5 g) were measured and 5 ml of a mixture of HCl and methanol solution (1:99 v/v) was added for extraction of anthocyanins (for 24 h at 4°C at dark). Each extraction solution was centrifuged at 3000 g for 30 min (MSE, Centaur 2, Sanyo, UK). After centrifugation, supernatant liquor was decanted and absorbance of extract was determined at 530 nm using a UV-2550 spectrophotometer. Based on the absorbance data, anthocyanin contents were calculated as U _(*A*530)_.g^−1^ FW (fresh weight).

### Small RNA library preparation and sequencing

Peels from debagged and unbagged “Granny smith” apple fruits were collected at 0, 6 h and 1 day, which were used for small RNA sequencing experiment. Total RNA was extracted from each pool, corresponding to the six sample groups, debagging (0, 6 h, 1 day) and unbagged samples (0, 6 h, 1 day); these six samples were denoted N1, N2, N3, T1, T2, and T3. The total RNA from each sample was extracted using TRIzol reagent (Invitrogen, USA) according to the manufacturer's instructions. Total RNA integrity was measured on an Agilent 2100 Bioanalyzer system (Agilent Technologies, Palo Alto, CA) for quality control. Samples of an RNA integrity number (RIN) > 8 were used for cDNA library preparations. The construction of peel sRNA libraries consisted of the following steps: (1) polyacrylamide gel electrophoresis (PAGE) purification of the RNA bands and RNA molecules in a size range of 16–35 nt enriched; (2) ligation of the 3p and 5p adapters to the RNA; (3) PCR amplification to generate a cDNA library for Illumina sequencing. These libraries were used for paired-end 45 × 2 sequencing using Illumina HiSeq™ 2000 at the Beijing Genomics Institute (BGI) (Shenzhen, China). Sequencing analysis was done using an Illumina Genome Analyzer (Illumina, CA, USA) according to manufacturer's instructions. Small RNA libraries sequencing was completed in genedenoveo company (Guangzhou, China).

### Small RNA analysis and miRNAs prediction

Raw data were pre-processed using common pipeline; clean reads were first used for bioinformatics analysis. Raw reads were filtered to remove low quality reads, ploy A, ≤ 18 bp reads and adaptor sequences. All clean reads were primarily mapped to the apple genome reference sequences by soap2 software (http://www.rosaceae.org/species/malus/malus_x_domestica/genome_v1.0p) to check their distribution. The sequences were subsequent searched in Rfam (http://rfam.sanger.ac.uk/) and Genbank databases. The sequences which matched to non-coding rRNAs, tRNAs, snRNAs, and snoRNAs were discarded. The remaining clean reads were used to search against miRBase database (version 21.0, http://www.mirbase.org/) for known apple miRNAs identification.

Unidentified sequences that did not match any above databases were further analyzed to predict novel miRNAs by using Mireap software.

### Differential expression analysis of miRNAs

To determine expression patterns of miRNAs among different treatments, the frequency of miRNA counts was normalized as transcripts per million (TPM). The fold-change between different treatments was calculated as: fold-change = log_2_ (treat/control). If |fold change|>1, the expression difference was considered as significant. A positive value indicated up-regulation of a miRNA, while a negative value indicated down-regulation. The pheatmap software was used to generate heatmap of miRNA expression level.

### Target gene prediction and functional annotation

Target genes of differentially-expressed miRNAs were predicted using patmatch software. Gene Ontology (GO) (http://www.geneontology.org/) enrichment analysis and Kyoto Encyclopedia of Genes and Genomes (KEGG) pathway analysis (http://www.genome.jp/kegg/) were performed to further probe the functions of target genes. For GO enrichment analysis and KEGG analysis, GO terms with a corrected-*p* ≤ 0.05 and KEGG pathway with *Q* ≤ 0.05 were considered as significant enrichment.

### Analysis of miRNAs and target genes by qRT-PCR

Total RNA was extracted from “Granny Smith” and “Starkrimson” apple peels in both treatment and control groups from 0, 1, 2, 4, and 6 days using Trizol RNA plant plus reagent (Tiangen). Total RNA and RT primers were utilized to amplify cDNA by reverse transcription, following miRcute miRNA First-Strand cDNA Synthesis Kit's instructions. Products were used as templates to analyze expression level of miRNAs following miRcute miRNA qRCR Detection Kit manufacturer's instruction.

Total RNA was used to detect the amount of expressed target genes. qRT-PCR was used to detect the expression level of target genes. Amplifications were carried out with Faststar Essential DNA Green Master (Roch) reagent in Biorad real-time PCR machine (iQ5.0, USA) following manufacturer's instructions. All reactions were repeated three times. 5S rRNA and actin gene were used as reference genes. All primers are listed in Table S1. The Expression levels of miRNAs and target genes were calculated by using the 2^−ΔΔCt^ method.

## Results

### Sequence analyses

**Figure 2:**
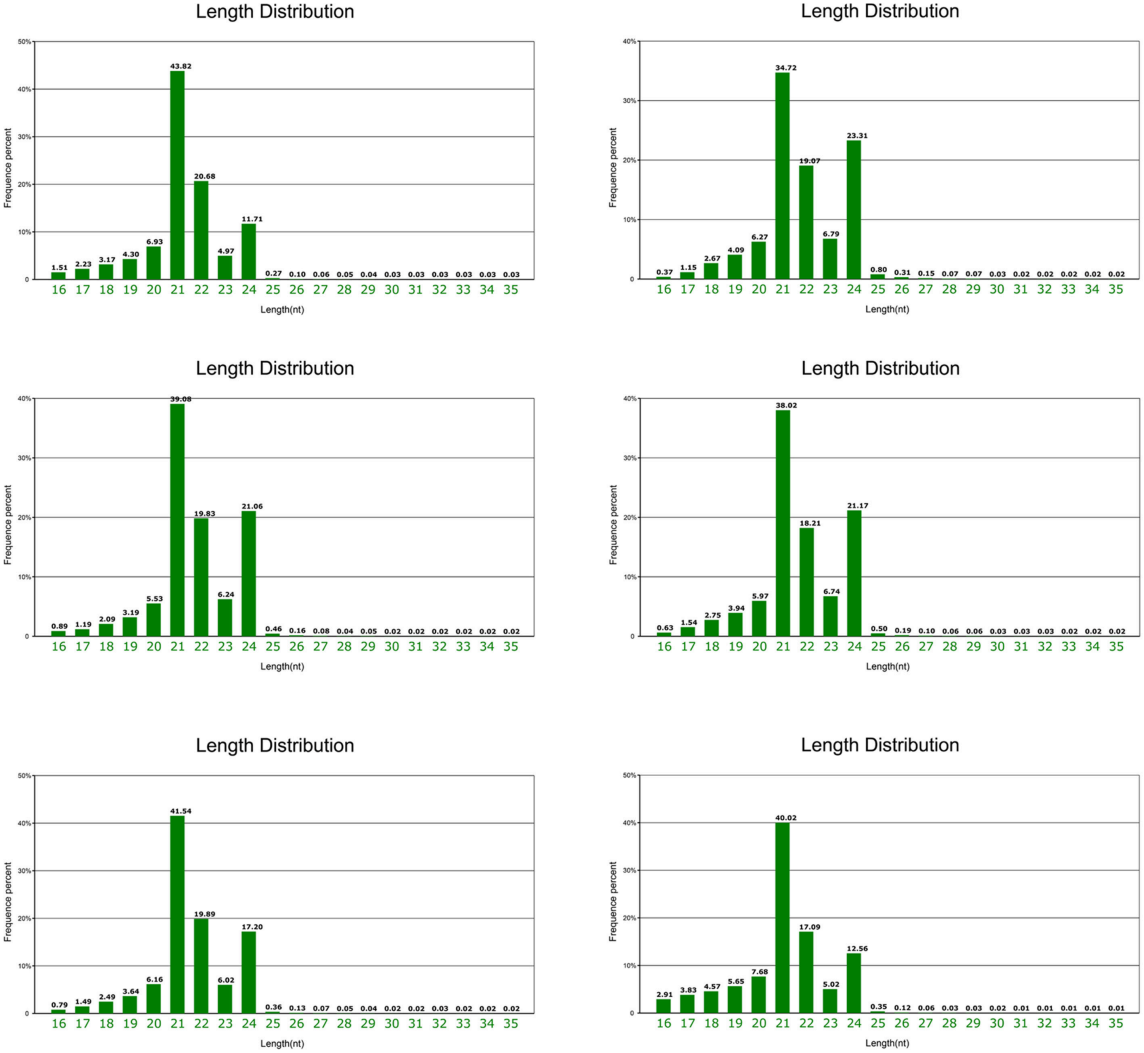
**Length and size distribution of unique sequences in six libraries from apple peel**.

Mature “Granny Smith” fruits peels from the debagging group (treatment) were collected at 0, 6 h, and 1 day after bag removal, and peels were also collected at the same time points in the unbagged group (control). Six small RNA (sRNA) libraries were constructed for deep sequencing. Total reads of 13,649,853(T1), 14,371,137(T2), 15,940,903(T3) 15,138,887(N1), 16,588,157(N2), and 15,980,780(N3), in six libraries, were generated from Illumina HiSeq, respectively (Table S2). Clean reads of 12,219,386(T1), 12,568,444(T2), 13,226,979(T3), 12,909,080(N1), 14,849,488(N2), and 14,140,213(N3) were obtained respectively after removal of the low-quality tags, 3′ adapter null, insert null, 5′ adapter contaminants, poly A reads and reads shorter than 18 nt (Table S2). Around 47% of clean reads in the six libraries were perfectly mapped to the apple genome with soap2 software, indicating that sequencing strategy did not create significant bias in these libraries. Various types of non-coding RNAs, including miRNA, rRNA, tRNA, snRNA, and snoRNA were then annotated (Table S2). The miRNAs were found to account for ~8.5% of the total sRNAs. sRNA from six libraries shared a similar distribution pattern, with 21 nt sRNAs being the most abundant, followed by 24, 22, and 23 nt, which is similar to size distribution of sRNAs in plants (Cao et al., 2014; Figure 2). However, this distribution pattern of sRNAs is different from that reported in other apple tissues, such as leaf, flower and root, with 24 nt sRNA being the majority (Xia et al., 2012).

### Identification of known miRNAs in apple fruit skin

**Figure 3:**
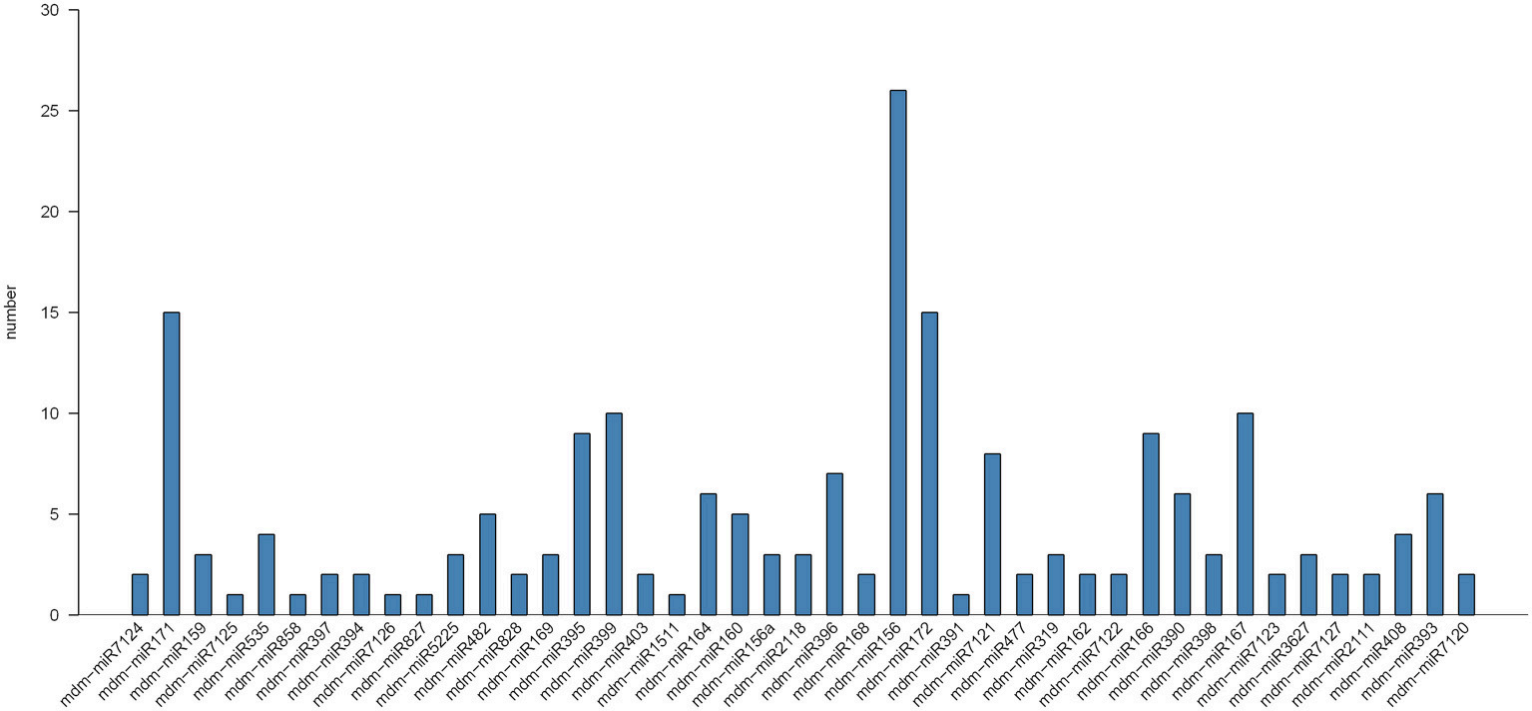
**Number of known miRNAs in different miRNA families in apple peel**.

To identify conserved miRNAs in mature apple peel, small RNA sequences were blast-searched against known plant miRNAs in miRBase database. Two hundred and one unique sRNA sequences were first identified, and they belonged to 43 apple miRNA families. Most of the identified miRNA families (75.12%) belonged to 21 nt-length miRNAs families, with the remaining belonging to 20 and 22 nt miRNAs families. Interestingly, the numbers of miRNA in each miRNA family were different. The largest miRNA family, miR156, had 29 members, followed by miR171 and miR172 with both 15 members (Figure 3). The expression levels of different members in each miRNA family were also significantly different (Table S3). For instance, in miR156 family, the read numbers of mdm-miR156a were 928, while the read numbers of mdm-miR156q were only 6. This large discrepancy of reads among miRNA family members might reflect their divergence of potential physiological roles during fruit development. Previous study shows that three miRNA families, MIR477, MIR482, and MIR3627, are highly conserved in fruit trees (Solofoharivelo et al., 2014). In our study, they were also found highly conserved in apple peel.

Apart from ones that perfectly matched to apple miRNAs, the remaining sRNA sequences were tentatively mapped to other plant miRNAs in miRBase database. Additional 108 unique reads with a minimum of 10 read counts were obtained in this case. They were conserved in a number of diverse plant species. Those highly-conserved apple miRNAs were also highly expressed in apple peel, including miR164 and miR393 with 398,744 and 261,279 reads in six libraries, respectively. In contrast the expression levels of less-conserved miRNAs were relatively low, such as miR827 and miR4388 with only 352 and 13 reads in six libraries, respectively.

### Identification of novel miRNAs in apple fruit skin

To search for potentially novel miRNAs in apple peel, Mireap software was used to explore the secondary structure of the novel miRNA sequences. Two hundred and twenty novel candidate miRNAs were predicted from six libraries (Table S4). The complementary miRNA^*^ sequences for each novel candidate miRNA were also detected, even though most were present at lower copy levels than those of their corresponding miRNAs. Furthermore, 30 out of 220 novel miRNAs were detected to have complementary miRNA^*^ sequences.

The length of the novel miRNAs ranged from 20 to 24 nt, among which 21 nt-length sequences were the most abundant (56%), followed by 23 nt- (22%), 22 nt- (18%), 20 nt- (4%), and 24 nt-lengh sequences (1%), which was consistent with typical length distribution of mature miRNAs. Compared with conserved miRNAs, most of the novel miRNAs were found to present at low copies, except novel-m0088-3p, novel-m0139-5p and novel-m0140-3p, which possessed more than 1000 normalized reads.

### Analysis of differentially expressed miRNAs

To explore the expression-level changes of miRNAs in “Granny Smith” subjected to bagging and debagging treatment, high-throughput sequencing analysis was performed to evaluate their expression level changes. Samples were collected at 0 h (bagged fruits grown under continuous dark before bag removal, T1), 6 h (fruits were subjected to 6 h sunlight after bag removal, T2) and 1 day (fruits grown 1 day under natural light conditions after bag removal, T3). Unbagged fruits (fruit grown under natural light conditions without any bagging treatment) were collected at 0 h (N1), 6 h (N2), and 1 day (N3) as controls. Only miRNAs with expression values higher than a 2-fold change were deemed significantly regulated.

In T1/N1, T2/N2, and T3/N3 groups, 87 known miRNAs (from 22 families) and 39 novel miRNAs (from 32 families) were found to be differentially expressed. The members of differentially expressed miRNAs were different in each group. For example, seven known miRNAs (miR160, miR164, miR395, miR477, miR5225, miR7125, and miR828) were present in all the three groups. While differentially-expressed miR172 and miR398 were only present in the T1/N1 group, miR167, miR391, miR7121, and miR858 were only in the T2/N2 group, and miR535 and miR7127 only in the T3/N3 group.

**Figure 4:**
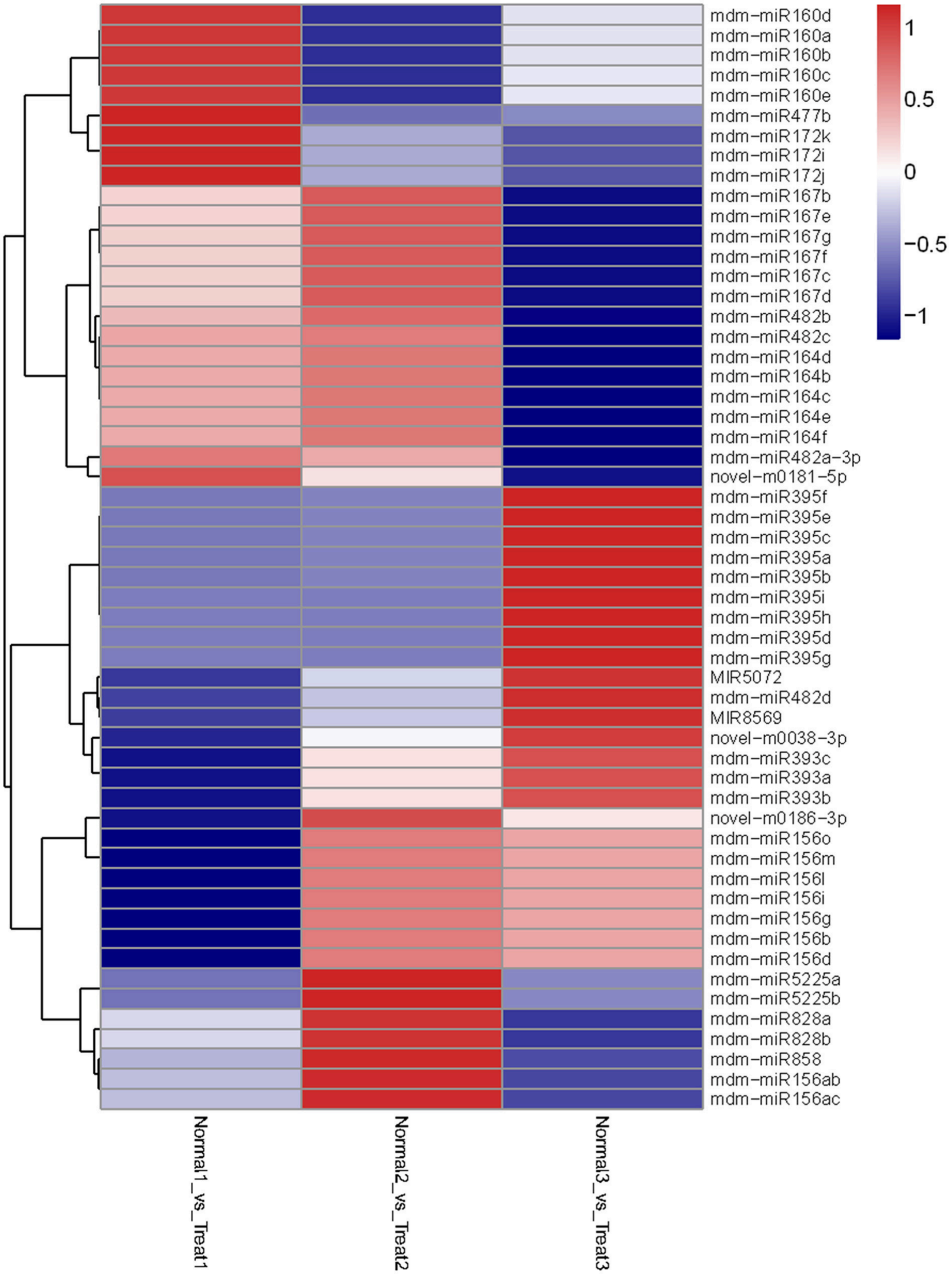
**Heatmap of differentially expressed miRNAs among N1/T1, N2/T2, and N3/T3 under bagging treatment**. Each column represents a stage, and color bar indicates relative expression level from high (red) to low (blue).

**Figure 5:**
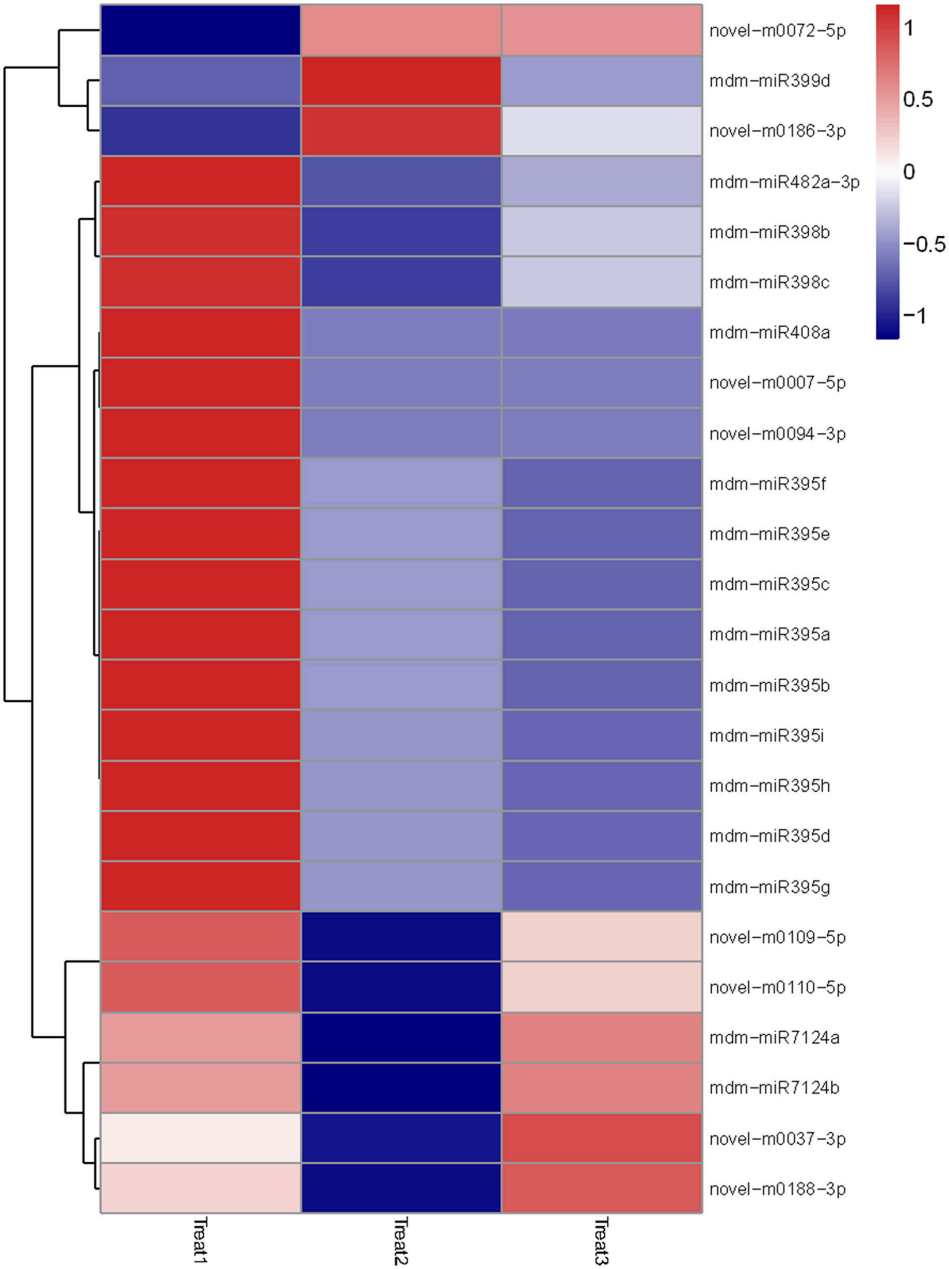
**Heatmap of differentially expressed miRNAs among T1, T2, and T3 after debagging treatment**. It shows expression levels of miRNAs. Each column represents a stage, and color bar indicates relative expression level from high (red) to low (blue).

Then we analyzed differentially-expressed miRNAs in the T1/N1 and T2/T1 groups. In the T1/ N1 group, 70 unique miRNAs sequences that significantly differentially expressed from 14 known and 15 novel miRNA families were detected (Figure 4). Compared with the expression levels of miRNAs in T1 (long term darkness), the expression levels of 47 miRNAs (67%) from N1 (natural sunlight conditions) were up-regulated, and the expression levels of the other 23 miRNAs (33%) were down-regulated. Moreover, the expression levels of differentially-expressed miRNAs among different members in one miRNA family showed different scales of change. For instance, the expression levels of miR399a-c and miR399i were down-regulated 3.6-fold and 6.5-fold, respectively. In T1/T2 group, 24 miRNAs were found to be significantly differentially-expressed, including 6 known and 8 novel miRNA families (Figure 5). The expression levels of miRNAs in T2 (re-exposed to sunlight 6 h) were significantly up-regulated compared to those of T1 (continuous darkness), except miR399d, novel-m0072-5p and novel-m0186-3p. The expression levels of miR398 in T2 significantly increased 4-fold compared to that in T1, followed by miR7124a-b, miR482a-3p, miR399d, miR408a, and miR395. The results indicated that the expression levels of some miRNAs were light-inducible.

The expression levels of miRNAs were also compared between unbagged and debagged samples. Of novel miRNAs identified, the expression levels of 32 novel miRNAs were shown to be up-regulated or down-regulated. The expression levels of most novel miRNAs were up-regulated in T1/N1 and T3/N3 groups. However, it was not observed in novel miRNAs of T2/N2 group, except two miRNAs (novel-m0081-5p and novel-m0092-3p). The results suggested that the expression levels of novel miRNAs were significantly changed in response to debagging treatment.

### Target prediction analysis of differentially expression miRNAs

To better understand functions of miRNAs in apple peel, it is necessary to identify their target genes. In this study, we obtained 1334 target mRNAs and 1055 target mRNAs for conserved miRNAs and novel miRNAs, respectively. More than one transcript was identified as a possible target gene for the majority of miRNAs. These predicted target genes belonged to a large number of transcription factors and functional gene families with different biological functions. They involved in various functions, including energy metabolism, RNA metabolism, starch and sucrose metabolism, signal transduction, secondary metabolite biosynthesis and stress responses. For example, ARFs (auxin response factors) were predicted to be modulated by miR160 and miR167. NAC transcription factors regulating fruit-ripening and anthranilate N-hydroxycinnamoyl/benzoyl transferase were shown to be regulated by miR164. AP2 (apetala2) transcription factors were the possible target genes of miR172. SPL (squamosa promoter-binding protein-like) transcription factors were predicted to act as the target genes of miR156. MdMYB transcription factors and anthocyanin regulatory C1 protein-like were found to serve as the target genes of miR828 and miR858. In addition, many functional genes were predicted to be the target genes of the differentially expressed miRNAs in our results. For instance, miR395 was predicted to target ATP sulfurylase and ubiquitin-conjugating enzyme E2; miR477 was found to target DELLA protein GAI1-like and pentatricopeptide repeat-containing protein; and miR5225 was predicted to target LRR receptor-like serine/threonine-protein kinase. The predicted target genes of miR482 were found to involve in NBS resistance protein, TMV resistance protein, and plant invertase and pectin methylesterase inhibitor superfamily. The target genes of the novel miRNAs also consisted of transcription factors and functional genes. For example, novel-m0038-3p and novel-m0181-5p were showed to target bHLH transcription factors and novel-m0186-3p target heat shock protein.

**Figure 6:**
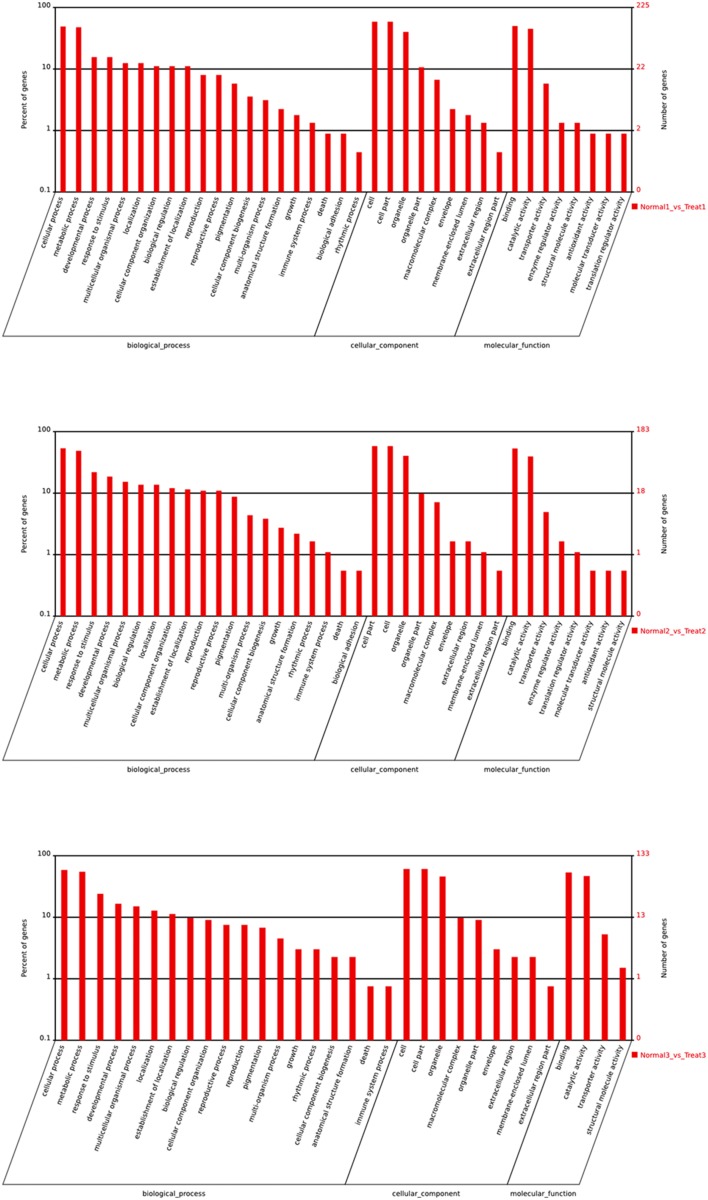
**GO analysis of differential expression miRNAs**.

To further gain insights into possible roles of differentially expressed miRNAs, putative target genes of differentially expressed miRNAs were subjected to GO analysis (Figure 6). GO categorization showed that target genes involved in the major biological processes were enriched in cellular processes, metabolic processes and responses to stimulus. The majority of genes were important in binding and catalytic activity. The functions of target genes, especially in T3/N3 group, involved in enzyme regulator, activity antioxidant activity and so on, indicating that differential expression of target genes was responsible for bagging and debagging treatment.

Kyoto Encyclopedia of Genes and Genomes (KEGG) database was also used to analyze the predicted target genes. Results showed that the target genes of differentially expressed miRNAs upon debagging treatment regulated secondary metabolism and hormone signal transduction, resulting in adaptive environment responses and fruit-ripening.

### qRT-PCR analysis of miRNAs and their target genes

**Figure 7:**
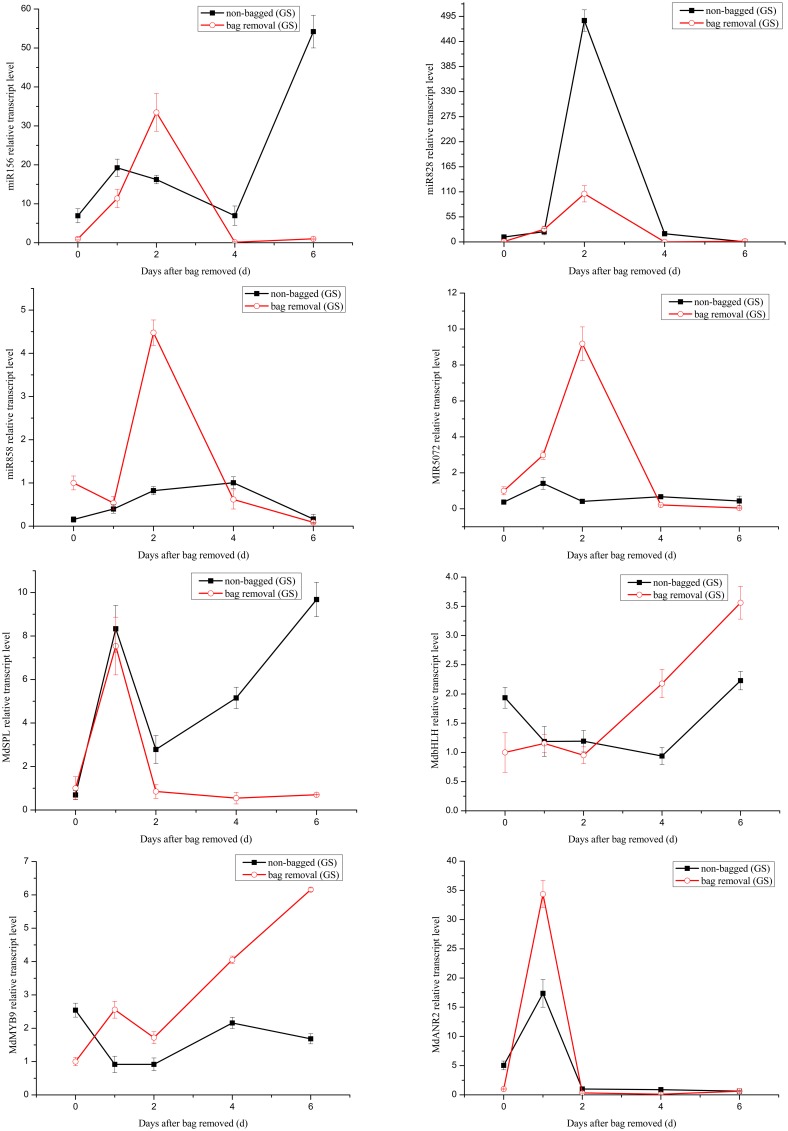
**Quantitative analysis miRNAs and expression levels of their target genes in “Granny Smith.”** Quantitative analysis of miRNAs and target genes expression level in “Granny Smith” apple peel by qRT-PCR at 0, 1, 2, 4, and 6 days. Error bars indicate SD obtained from three biological replicates.

**Figure 8:**
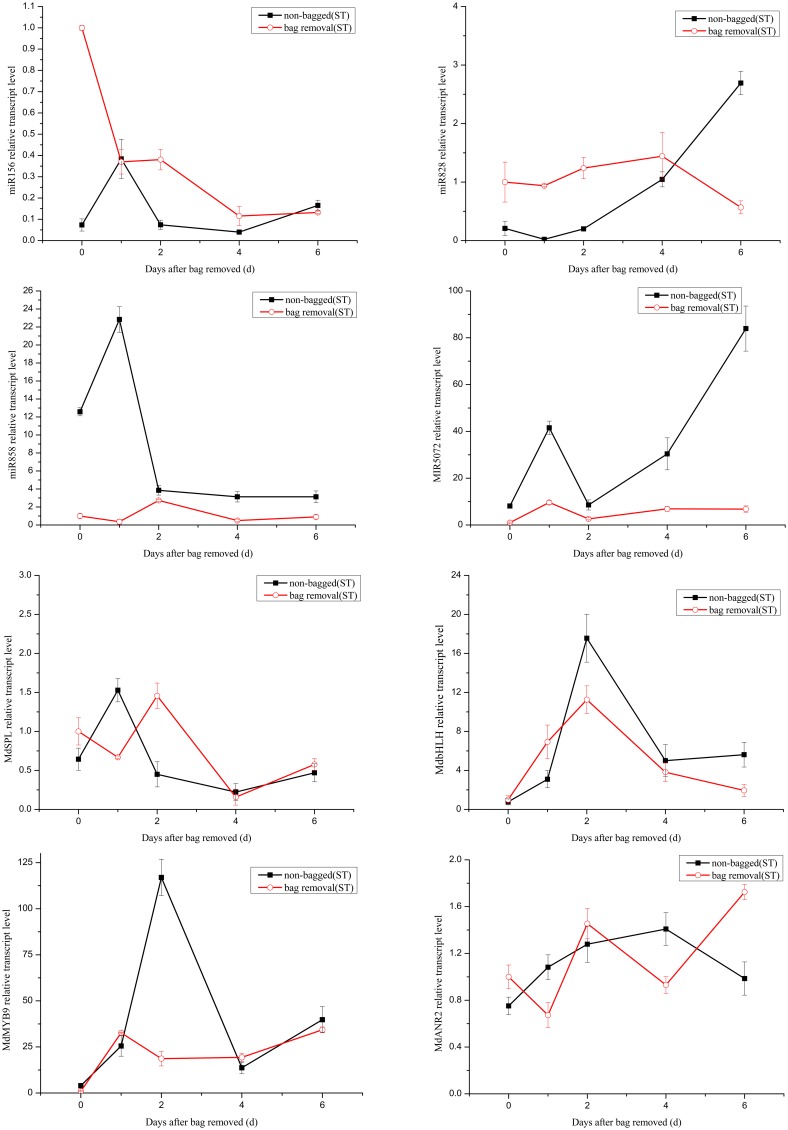
**Quantitative analysis miRNAs and expression levels of their target in “Starkrimson.”** Quantitative analysis of miRNAs and target genes expression level in “Starkrimson” apple peel by qRT-PCR at 0, 1, 2, 4, and 6 days. Error bars indicate SD obtained from three biological replicates.

It has been reported that the expression level changes of miRNAs can influence the accumulation of anthocyanin (Xia et al., 2012). Anthocyanin concentrations in red cultivar “Starkrimson” and green cultivar “Granny Smith” significantly increased after debagging in our study (Figure S1). Our attention was thereby focused on the potential roles of miRNAs in regulating anthocyanin accumulation during debagging. The expression levels of four known miRNAs (mdm-miR156, mdm-miR828, mdm-miR858, and miR5072) and their target genes were investigated by using qRT-PCR (Figures 7, 8).

The transcription levels of mdm-miR156 in “Granny Smith” after debagging were very low at 0 day, and then continuously increased to the maximum at 2 days, followed by a prominent decrease till 6 days. Meanwhile the expression levels of miR156 in controls were sharply increased at 1 day and decreased to the minimum at 4 days, and increased again to the maximum at 6 days. However, in treatment group of “Starkrimson,” the expression levels of miR156 continuously decreased from 0 h to 6 days and in the “Starkrimson” control group, the expression levels of miR156 were slightly fluctuated at low expression level. The expression levels of mdm-miR828 in treatment and control groups of “Granny Smith” sharply increased and reached to highest level at 2 day, then both of them dramatically decreased to undetectable level at 6 days. The expression levels of “Starkrimson” mdm-miR828 in the treatment group were only slight increased to their highest level at 4 days, and then decreased at 6 days. In the control group, the transcription level of mdm-miR828 gradually increased from 0 h to 6 days. The mRNA abundances of mdm-miR858 and miR5072 in the “Granny Smith” control group and the “Starkrimson” treatment group were slightly fluctuated at low expression level. The expression levels of “Granny Smith” mdm-miR858 in the treatment group were decreased at 1 day, and then rapidly up-regulated at 2 days, finally down-regulated to lowest level at 6 days. In contrast, those of “Starkrimson” in control increased at 1 day, followed reduced to minimum value at 6 days. The expression levels of “Granny Smith” miR5072 in the treatment group were rapidly up-regulated at 2 days, and then down-regulated to lowest level at 6 days. The expression levels of “Starkrimson” miR5072 in control increased at 1 day, followed reduced to minimum value at 2 days and then increased again till 6 days.

Predicted target genes of differential expression miRNAs were used to elucidate the relationship between functions and phenotypes. The expression profiles of *MdSPL9* (MDP0000297978), *MdbHLH* (MDP0000225680), *MdMYB9* (MDP0000210851), and *MdANR2* (MDP0000320264) target genes regulated by mdm-miR156, mdm-miR828, mdm-miR858 and miR5072 were analyzed, respectively.

The changes of mRNA abundance of *MdSPL9* gene in treatment group of “Granny Smith” were sharply increased at 1 day and then decreased to minimum, while those in control group were sharply increased at 1 day and then decreased at 2 days, followed continuously increasing. The transcription levels of *MdSPL9* gene in the “Starkrimson” treatment and control groups were gradually decreased, except a 1.5-fold increase at 1 day in control group and a 1.4-fold increase at 2 day in treatment group. The transcription levels of *MdbHLH* gene in “Granny Smith” slightly fluctuated and then remarkably increased at 2 and 4 days, in treatment and control groups, respectively. However, the expression levels of *MdbHLH* gene in debagging and unbagged groups of “Starkrimson” increased to the maximum at 2 day, and then rapidly decreased. The expression levels of *MdMYB9* gene in treatment group were up-regulated in both cultivars, with a sharply increase in “Granny Smith” after debagging. The transcription levels of *MdANR2* in treatment group were decreased in “Granny Smith,” while increased in “Starkrimson.”

## Discussion

In the Loess Plateau region of China, it is interesting that the bagged green cultivar “Granny Smith” can turn red after debagging during ripening (Figure 1A). Various biochemical and molecular biology studies have been performed to probe the coloration mechanisms of “Granny Smith” in response to the debagging treatment. Previous studies were mainly performed at metabolic and transcriptional levels (Liu et al., 2013; Wang et al., 2013; Zhang et al., 2013; Meng et al., 2015), reports at the post-transcriptional level are still not available, even though miRNAs have been shown to play pivotal roles in post-transcriptional levels (Jeong and Green, 2013; Inal et al., 2014; Kumar, 2014; Wu et al., 2014; Rosas-Cárdenas et al., 2015). Apple miRNAs of different tissues had been identified by using computational and/or sequencing approaches (Varkonyi-Gasic et al., 2010; Xia et al., 2012; Ye et al., 2013; Yu et al., 2014). However, whether miRNAs are responsible for debagging treatment is still unknown. Therefore, identification of apple miRNAs and associated target genes in response to debagging will improve our understanding of molecular regulatory mechanisms of apple skin coloration.

### Overview of the deep-sequencing datasets

In this study, we performed high-throughput sequencing of “Granny Smith” apple peel libraries, constructed from both debagging (at 0, 6 h, and 1 day) and unbagged treatments (at 0, 6 h, and 1 day). High quality sequencing reads were 16–35 nt in length, a wide range of sRNAs compared to 17–26 nt in a previous report (Figure 2; Visser et al., 2014). In addition, the percentage of 21 nt sRNAs (averaging 39.52%) was much higher than that of 24 nt sRNAs (averaging 17.83%) in apple peel. The result was consistent with the length distribution of sRNAs found in strawberry, orange and *Brassica juncea* (Li et al., 2013; Yang et al., 2013; Zhang et al., 2014b). However, our result was different-length from those reported in other apple tissues, where 24 nt sRNA is most abundant in leaf, root, flower and young fruit (Xia et al., 2012). The distribution of different length sRNAs may be attributed to different tissue types and/or different apple cultivars. Therefore, it is suggested that miRNAs play diverse roles in apple development.

Comprehensive miRNAs sequences analysis revealed 201 mature known mdm-miRNAs from 43 miRNA families and 220 novel miRNAs in the six apple peel libraries. Analysis of the conserved miRNAs showed that mdm-miR393, mdm-miR397 and mdm-miR408 families were only present in mature fruits, but not in young fruits (Xia et al., 2012), suggesting that different miRNAs were expressed at different fruit development stages. Our result showed that miR482 had the highest read count in mature fruit, while miR396 is reported as the most abundance in young fruit (Xia et al., 2012), suggesting that miR482 and miR396 may play different roles during fruit development. It can be seen that the expression levels of miRNAs may be different at different development stages and various environmental conditions.

### Analysis of differentially expressed miRNAs

The expression levels of miRNAs were found to be significantly changed after debagging. Compared with the unbagged group, 67% differentially expressed miRNAs were up-regulated in the bagged group (T1/N1). After 6 h debagging (T2/T1), almost all differentially-expressed miRNAs were significantly up-regulated, including miR395a-i, miR398b-c and novel-m0037-3p were up-regulated 3.5-fold, 4-fold and 5.9-fold, respectively. The result suggests that expression levels of most miRNAs in apple peel are light-inducible.

Previous studies have shown that the expression levels of several miRNAs can be regulated by different light conditions. For example, the expression of gma-miR167 is induced by far-red light (Jones-Rhoades and Bartel, 2004; Zhang et al., 2014a), the expression levels of ath-miR408 is induced by light (Li et al., 2014; Zhang et al., 2014a), and the expression levels of ath-miR396 (Zhou et al., 2007), ptc-miR168a-b, ptc-miR169, and ptc-miR398 (Jia et al., 2009) are induced by UV-light. In our results, some miRNAs were found to be differentially regulated when bagged fruits were re-exposed to sunlight. For example, we found that miR160, miR156, miR171, miR172, miR395, and miR398 were differentially expressed upon debagging, these families are predicted to be regulated by UV-B radiation in *Arabidopsis* and juvenile maize leaves, respectively (Zhou et al., 2007).

Previous studies show that fruits are very sensitive to high light after debagging (Li and Cheng, 2008a; Chen et al., 2012), and direct solar radiation can result in photo-oxidation in apple peel (Li and Cheng, 2008b). In our results, we observed the same responses of miRNAs and their associated target genes to high light stress. For example, HBCT (anthranilate N-hydroxycinnamoyl and benzoyltransferase) related to phytoalexin biosynthesis was shown to serve as target gene of miR164 (Han et al., 2011) and the *HBCT* gene plays an important role in protection against high light stress by activating phytoalexin synthesis. The *HBCT* gene has been confirmed as cultivar-specific genes in the green cultivar “Greensleeves” but not in the red cultivar “Redfield” in a previous report (Han et al., 2011). It has not been reported in “Granny Smith.” In our results, the expression levels of miR164 in “Granny Smith” decreased after debagging; this could increase expression level of *HBCT* gene and protect apple peel from light stress. In addition, our results showed fruits could be subjected to oxidative stress after debagging. Regulation of autophagy was predicted after debagging, which can minimize damage and/or remove superfluous proteins in order to cope with oxidative stress. GO analysis revealed that the target genes of the differentially expressed miRNAs were related to DNA photolyase activity and oxidoreductase activity in T3/N3 group. KEEG analysis revealed that they were involved in antioxidant systems in apple peels, including flavonoid biosynthesis, arachidonic acid metabolism, ascorbate and aldarate metabolism, glutathione metabolism and peroxisome; similar results were also reported in previous studies (Wünsche et al., 2001; Felicetti and Schrader, 2008). Since flavonoid biosynthesis plays an important role in protection against UV radiation in plants (Smith and Markham, 1998; Ryan et al., 2001; Solovchenko and Schmitz-Eiberger, 2003), our results indicated that apple peel could enhance its photo-protective capacity to cope with light stress from 0 h to 1 day after bag removal (Zhang et al., 2015).

The increase of anthyocanin concentrations in apple peel after debagging is another indication of a photo-protective role (Figure S1). Anthocyanin accumulation can play photo-protective roles in plants under high light stress. It has been reported that miR828 and miR858 could directly or indirectly control anthocyanin biosynthesis in apple, and differentially expressed miR156 can positively regulate anthocyanin biosynthesis by the SPL transcription factor in *Arabidopsis thaliana* (Gou et al., 2011; Xia et al., 2012). Interestingly, our results found that the expression levels of mdm-miR156, mdm-miR828 and mdm-miR858 were different when fruits were debagged. These differentially expressed miRNAs and their associated target genes might regulate anthocyanin biosynthesis after debagging. It can be seen that anthocyanin accumulation in apple could be regulated by miRNAs subjected to environment stress after bag removal.

Many differentially-expressed miRNAs were modulated in response to heat stress. Differentially-expressed miR398 is very sensitive to heat stress. Heat induced miR398 can trigger a regulatory loop which is critical for thermal tolerance in *Arabidopsis* (Guan et al., 2013).

### Related to anthocyanins biosynthesis miRNAs

Anthocyanin contributes to red coloration in red apple skin. Sunlight is a significant factor promoting anthocyanin production in apple peel, especially for apple fruits subjected to bagged and debagging treatments (Takos et al., 2006). Our study clearly showed that anthocyanin concentrations were extremely low in bagged “Granny Smith” peel and bagged “Starkrimson” peel. However, after debagging, anthocyanin concentrations significantly increased in both cultivars. Red cultivar “Strakrimson” developed dark red color with higher anthocyanin concentrations after debagging (Meng et al., 2015), and the green cultivar “Granny Smith” skin color also turned red (Figure 1). Previous reports indicate that the higher expression of regulatory genes can lead to the accumulation of anthocyanin in “Granny Smith” (Wang et al., 2013). In this study, few candidate miRNAs and their target genes were found to be associated with regulation genes of anthocyanin biosynthesis in “Granny Smith.”

Target genes of miR156 have been known as SPL transcription factors in *Arabidopsis*, which are exclusively expressed in the shoot apex (Cardon et al., 1999; Schwarz et al., 2008). It has been reported that miR156 can positively regulate anthocyanin biosynthesis by SPL transcription factor, and SPL transcription factor can negatively regulate accumulation of anthocyanin through destabilization of a MYB-bHLH-WD40 complex (Gou et al., 2011). In our study, after 2 day of sunlight re-exposure, the expression levels of MdSPL transcription factor in the “Granny Smith” treatment group was lower than that of the control group. Therefore, the increased activity of MYB-bHLH-WD40 complex in the treatment group leaded to the accumulation of anthocyanin. Similarly, the transcription levels of MdSPL transcription factor in “Starkrimson” were gradually decreased after bag removal, suggesting an increased anthocyanin level. In addition, the expression of target gene *MdSPL* was a perfect inverse to that of mdm-miR156 after 1–2 days debagging in “Granny Smith.” However, the change trends of miR156 and its target gene were rather similar in “Starkrimson.” Therefore, the results showed that mdm-miR156 could promote the accumulation of anthocyanin by MdSPL transcription factors in “Granny Smith” after debagging, but not in “Starkrimson.”

*MdTAS4-siR81*(-), derived from the miR828-TAS4 pathway, has three predictable *MYB* target genes. MdbHLH3 transcription factor, regulating anthocyanin biosynthesis in apple, is one of *MdTAS4-siR81*(-) target genes (Xia et al., 2012). In our study, we found several target genes of miR828 were related to anthocyanin biosynthesis in apple peel after debagging. Among them, the expression of *MdbHLH* gene was a perfect inverse to that of mdm-miR828 in both cultivars. Therefore, MdbHLH transcription factor was one target gene of mdm-miR828. The expression levels of *MdbHLH* gene in “Granny Smith” slightly fluctuated at 2 day after debagging and then continually increased from 2 to 6 days debagging, which was similar to the changes of anthocyanin concentration (Figure 2). Moreover, the changes of *MdbHLH* mRNAs abundance in “Starkrimson” were higher than those in “Granny Smith.” It can be inferred that mdm-miR828 and *MdbHLH* gene could have an important influence in regulating the accumulation of anthocyanin in the two cultivars.

Previous study shows that miR828 is specifically expressed in apple flowers, but miR858 accumulates most abundantly in mature apple fruit (Xia et al., 2012), in agreement with our results that the normalized reads of miR858 were 120-fold higher than that of miR828. It has been indicated that MdMYB9 transcription factor can bind to *ANS, ANR*, and *LAR* promoters, thereby promoting accumulation of anthocyanin and proanthocyanin (PA) in apple calluses (Gesell et al., 2014). Interestingly, in our study, MdMYB9 transcription factor was predicted to be targeted by mdm-miR858. We found that the expression of miR858 actually reversed to MdMYB9 transcription factor, implying that mdm-miR858 might be negatively correlated with the accumulation of anthocyanin and PA in apple peel. Moreover, the transcription levels of MdMYB9 transcription factor in “Granny Smith” sharply increased at 2 day after debagging, which was largely concordant with the trend of anthocyanin accumulation. It was suggested that the regulation of mdm-miR858/*MdMYB9* might be involved in anthocyanin biosynthesis in “Granny Smith” apple peel after debagging. Although the expression levels of mdm-miR858 slightly fluctuated in the “Starkrimson” treatment group, transcription levels of MdMYB9 transcription factor increased to 40-fold at 6 days after debagging, which was in agreement with trends of increasing anthocyanin concentrations. It can be seen that transcription level of MdMYB9 transcription factor caused by mdm-miR858 was more readily influenced in “Starkrimson” than that in “Granny Smith,” and mdm-miR858 played an important role in anthocyanin accumulation in both cultivars. In addition, previous studies have demonstrated that MdbHLH transcription factor can bind to *MdMYB9* promoters and regulates its transcription *in vitro*, which is consistent with our results that the expression levels of MdbHLH and MdMYB9 transcription factors in “Granny Smith” began to sharply increase at 2 days after debagging, resulting in anthocyanin accumulation.

Target gene *MdANR2* (anthocyanidin reductase) was regulated by miR5072. Three genes encoding *ANR*, designated *MdANR1, MdANR2a*, and *MdANR2b*, have been characterized. Ectopic expression of apple *MdANR* genes in tobacco flower positively and negatively regulate PAs and anthocyanin biosynthesis, respectively (Han et al., 2012). In our results, the expression of target gene *MdANR* was a perfect inverse to that of miR5072 in “Starkrimson,” but not in “Granny Smith.” Therefore, the results showed that miR5072 only promotes the accumulation of anthocyanin by *MdANR* gene in “Starkrimson” after debagging. The expression levels of *MdANR* gene in “Starkrimson” were up-regulated after debagging, resulting in anthocyanin accumulation.

These four differentially expressed miRNAs are therefore believed to regulate the accumulation of anthocyanins in apple peel with debagging treatment. Among them, mdm-miR828 and mdm-miR828might play important roles in the accumulation of anthocyanin after debagging in “Granny Smith” and “Starkrimson” apple cultivars. As for mdm-miR156 in “Granny Smith,” and miR5072 in “Starkrimson,” they can increase anthocyanin concentration upon bag removal.

## Author contributions

ZZ and DQ designed this research. DQ and FY performed the experiments, data analysis and wrote the manuscript. RM, XJ, HY, and ZG helped bagged apples and ready for plant marterial. YD helped modify the manuscript.

### Conflict of interest statement

The authors declare that the research was conducted in the absence of any commercial or financial relationships that could be construed as a potential conflict of interest.

## Supplementary material

Figure S1 **Anthocyanin accumulation in apple skin of “Granny Smith” and “Starkrimson” after debagging**. Error bars indicate SD obtained from four biological replicates.

Table S1 **List of primers used for qPCR analysis**.

Table S2 **Composition of small RNA sequences from N1, N2, N3 and T1, T2, T3 libraries of the apple peel**.

Table S3 **List of differentially expressed miRNAs**.

Table S4 **List of novel miRNAs**.
