# Supplementary figures and images for: Identification of MicroRNAs and Their Targets Associated with Fruit-Bagging and Subsequent Sunlight Re-exposure in the “Granny Smith” Apple Exocarp Using High-Throughput Sequencing
Source: Front Plant Sci. 2016 Feb 1;7:27. doi: 10.3389/fpls.2016.00027 (PMC4734179; doi:10.3389/fpls.2016.00027)

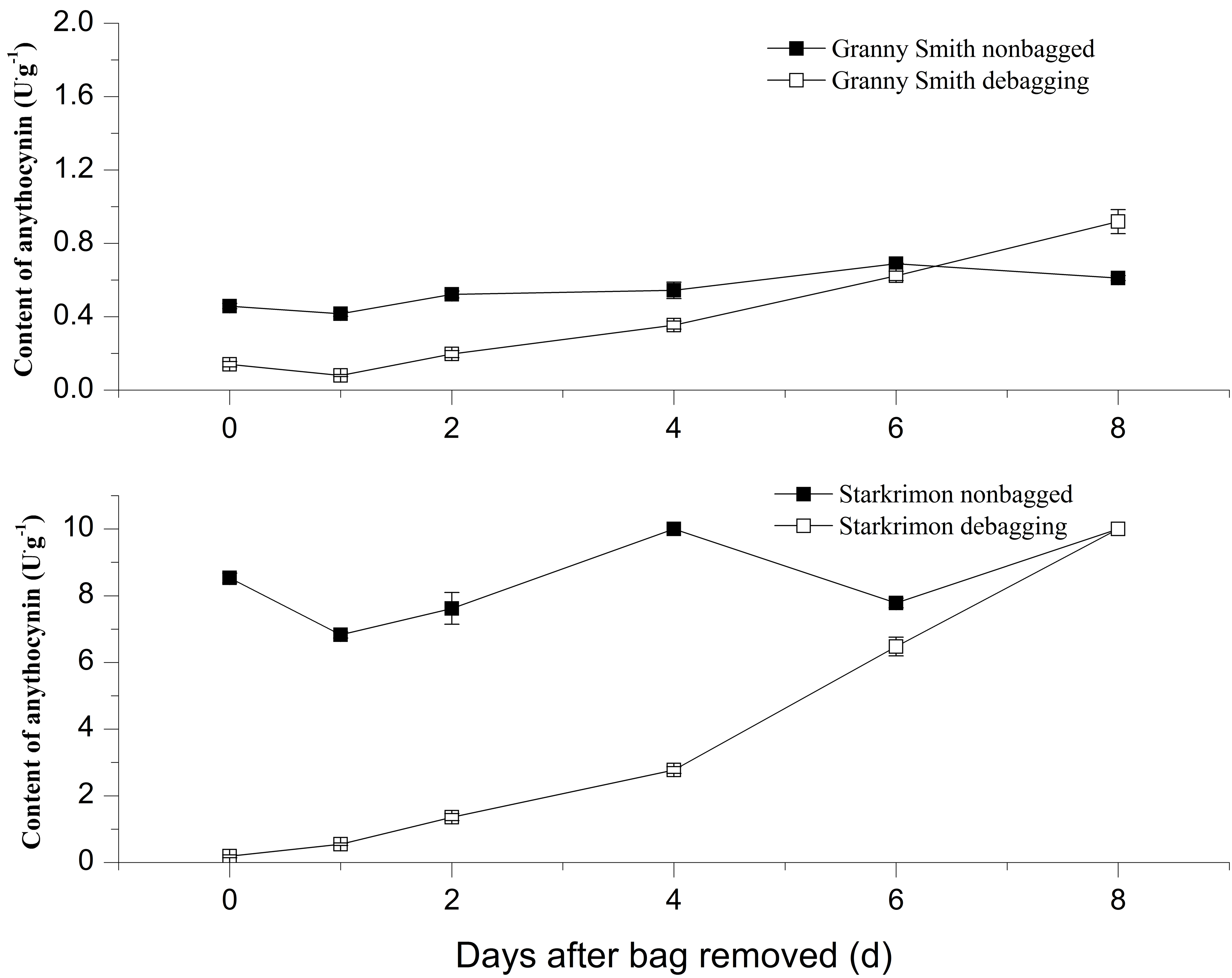

Supplement: Figure S1 — Anthocyanin accumulation in apple skin of “Granny Smith” and “Starkrimson” after debagging. Error bars indicate SD obtained from four biological replicates. [file Image1.TIF]
