# Supplementary material for: Identification of MicroRNAs and Their Targets Associated with Fruit-Bagging and Subsequent Sunlight Re-exposure in the “Granny Smith” Apple Exocarp Using High-Throughput Sequencing
Source: Front Plant Sci. 2016 Feb 1;7:27. doi: 10.3389/fpls.2016.00027 (PMC4734179; doi:10.3389/fpls.2016.00027)
Supplement: Table S1 — List of primers used for qPCR analysis. [file Table1.DOC]

**S1 Table. Forward and reverse primers used in qPCR analysis of candidate miRNAs and target genes.**

| Gene name | Forward primer(5’-3’) | Reverse primer (5’-3’) | Gene ID |
| --- | --- | --- | --- |
| mdm-miR156 | GACAGAAGATAGAGAGCACA |  |  |
| mdm-miR828 | CGCTCTTGCTCAAATGAGTATTCCA |  |  |
| mdm-miR858 | TTCGTTGTCTGTTCGACCTGA |  |  |
| miR5072 | TCCCCAGCAGAGTCGCCA |  |  |
| MdSPL9 | GACCTCCAATCACTTTCCG | TTCATCTGCTGACTGGTGG | MDP0000297978 |
| MdMYB9 | ACATTGATGCGAAGGGAG | TAAATCCAACGAGGCACC | MDP0000210851 |
| MdbHLH | AGGGTTCCAGAAGACCACGCCT | TTGGATGTGGAGTGCTCGGAGA | MDP0000225680 |
| MdANR2 | TCCCAGCCACCTTTCCAGTTCA | ACCAGCAGAGACGCCACGAA | MDP0000320264 |
| 5s rRNA | GATCCCATTCCGACCTCGATATGTG | TTCAGTACATATGGCGCAAGACG | 13630181 |
| MdActin | TGACCGAATGAGCAAGGAAATTACT | TACTCAGCTTGGCAATCCACATC | CN938023 |
